# Integrating Clinical and Translational Research Networks—Building Team Medicine

**DOI:** 10.3390/jcm9092975

**Published:** 2020-09-15

**Authors:** Ravi Salgia, Prakash Kulkarni

**Affiliations:** City of Hope National Medical Center, Duarte, CA 91010, USA; pkulkarni@coh.org

In the United States (US), medical centers are widely recognized as vital components of the health care system. In general, however, the academic medical centers are differentiated from their community counterparts by their mission which typically focuses on clinical care, education, and research. Nonetheless, community clinics/hospitals fill a critical need and play a complementary role serving as the primary sites for health care in most communities. Furthermore, new health care reform initiatives in the US and economic pressures have created opportunities and incentives for hospitals and health systems to integrate, resulting in a nationwide trend toward consolidation. As a result, academic medical centers are leveraging their substantial assets to merge, acquire, or establish partnerships with their community peers with the ultimate goal of ensuring that all patients, regardless of their physical proximity to major medical institutions, can benefit from recent clinical advances. 

This trend is highly pervasive across medical specialties, and as these alliances accelerate, they will continue to affect the oncology groups providing services at these institutions in particular. Thus, we believe a deeper understanding of the new landscape, changing relationships, and marketplace dynamics will help both academic and private practice oncologists adapt to this ongoing change. At the City of Hope, a United States National Cancer Institute (NCI)-designated Comprehensive Cancer Center, the leadership, working closing with the Chair of Medical Oncology and others, was swift in recognizing this challenge and executing a game plan to ensure that the best medical care developed and practiced by its academic center can be accessed by cancer patients throughout the enterprise, especially within the community centers. With >75 faculties in the Department of Medical Oncology, and 27 community centers in Southern California, we believe this is a sizeable enterprise in which we implemented this approach to integrate the academic and community centers. Thus, we trust that it would be valuable to share our experience with other healthcare providers and organizations in the US and other parts of the world so that they can benefit from our experience.

It is now increasingly recognized that in addition to physicians, physician-scientists, and other healthcare-related professionals, basic research scientists also contribute significantly to the emerging inter- and cross-disciplinary, team-oriented culture of translational science. Therefore, approaches that combine the knowledge, skills, experience, expertise, and visions of clinicians in academic medical centers and their affiliated community centers and hospitals, together with basic research scientists, are critical in shaping the emerging culture of translational research so that patients from the urban as well as suburban settings can avail the benefits of the latest developments in science and medicine.

This Special Issue is an embodiment of this ethos. It includes a series of papers authored by teams of leading clinicians, basic research scientists, and translational researchers. The authors discuss how engaging and collaborating with community-based practices, where the majority of older patients with cancer receive their care, can ensure that these patients receive the highest-quality, evidence-based care. Based on our collective experience, we would like to stress that the success of academic-community collaborative programs not only depends on the good will and vision of the participants but also on the medical administration, academic leadership, policy makers who define the principles and rules by which cooperation within the health care industry occurs. We refer to this cooperation and collaboration as ‘Team Medicine’. 

We take this opportunity to thank the City of Hope leadership for the support and encouragement; the authors for taking time from their hectic schedules, especially during this unprecedented pandemic to share their unique experience, vision, and ideas; and the patients and their families for their participation and enduring spirit. We trust that our experience embodied in this singular compendium will serve as a ‘Rosetta Stone’ for other institutions and practitioners.

